# Non-invasive estimation of inspiratory muscle pressure and work of breathing by airway pressure extrapolation from the P0.1 maneuver during assisted ventilation

**DOI:** 10.1038/s41598-026-61929-1

**Published:** 2026-07-16

**Authors:** Friederike Behmüller, Tatiana Maria Bastian, Helene Selpien, Christine Eimer, Norbert Weiler, Dirk Schädler, Giacomo Bellani, Tobias Becher

**Affiliations:** 1 Department of Anesthesiology and Operative Intensive Care Medicine, University Medical Center Schleswig-Holstein, Campus Kiel, Kiel, Germany; 2 Centre for Medical Sciences - CISMed, University of Trento, Trento, Italy; 3 Department of Anesthesia and Intensive Care, Santa Chiara Hospital, Trento, Italy

**Keywords:** Acute hypoxemic respiratory failure, Assisted ventilation, Inspiratory muscle pressure, Work of breathing, P0.1, Esophageal pressure, Diseases, Health care, Medical research, Physiology

## Abstract

Monitoring inspiratory effort during assisted mechanical ventilation is essential to balance lung protection and respiratory muscle loading. Esophageal pressure monitoring remains the reference standard for estimating inspiratory muscle pressure and work of breathing but is invasive and rarely used in routine clinical practice. Non-invasive alternatives based on routinely available ventilator signals are therefore needed. This monocentric physiological substudy of the ICEBERG observational trial included adult patients with acute hypoxemic respiratory failure undergoing assisted mechanical ventilation. Inspiratory muscle pressure (P_mus_) was first estimated non-invasively by linear extrapolation of the airway pressure drop recorded during a standardized end-expiratory occlusion (P0.1 maneuver) into early inspiration (up to 500 ms after occlusion). Using the extrapolated P_mus_ together with airway pressure, airway resistance was calculated during early inspiration, both with and without correction for respiratory system elastance determined by occlusion maneuvers. The resulting resistance and elastance were then used to parameterize the equation of motion of the respiratory system, allowing subsequent reconstruction of the inspiratory P_mus_ waveform over the entire breath. From the reconstructed pressure signal, peak inspiratory P_mus_, work of breathing, and pressure–time product were calculated. Esophageal pressure–derived measurements served as the reference standard, and agreement was assessed using linear regression and Bland–Altman analysis. Eighteen patients (age 67 ± 12 years, PaO₂/FiO₂ 198 ± 44 mmHg) contributed 33 valid measurements. Based on a patient-average analysis, non-invasively estimated P_mus_ during early inspiration showed statistically significant correlations with esophageal pressure–derived values across all analyzed time points (all p < 0.01), with the highest agreement and lowest bias observed at 100 ms extrapolation time after the end of the P0.1 maneuver. Elastance-corrected airway resistance demonstrated slightly improved agreement compared with uncorrected resistance estimates. Work of breathing derived from non-invasive inspiratory P_mus_ showed good agreement with reference measurements (R^2^ = 0.70; bias =  − 0.05 J), whereas pressure–time product exhibited greater bias and wider limits of agreement. A P0.1-guided, non-invasive method based on airway pressure extrapolation allows feasible and physiologically meaningful estimation of inspiratory P_mus_ during assisted ventilation. Derived work of breathing shows good agreement with esophageal pressure-based measurements, supporting the potential of this approach for non-invasive monitoring of inspiratory effort in patients with acute hypoxemic respiratory failure.

**Supplementary Information:**

The online version contains supplementary material available at 10.1038/s41598-026-61929-1.

## Introduction

Monitoring inspiratory effort during assisted mechanical ventilation is a central challenge in the management of acute hypoxemic respiratory failure (AHRF)^1^. In many patients, preservation of some degree of spontaneous breathing improves ventilation distribution and ventilation-perfusion matching^2^. Excessive inspiratory effort, however, may lead to diaphragmatic dysfunction and aggravate lung injury^3,4^, while insufficient effort promotes diaphragmatic atrophy, potentially leading to prolonged mechanical ventilation^5,6^. Achieving an optimal balance between lung protection and adequate respiratory muscle loading may therefore require quantification of inspiratory effort during assisted ventilation.

Inspiratory muscle pressure (P_mus_) represents the pressure generated by respiratory muscles to overcome elastic and resistive loads of the respiratory system. In conjunction with derived parameters such as work of breathing (WOB), P_mus_ provides quantitative information on respiratory muscle load and mechanical stress applied to the lung^7^. Esophageal pressure (P_es_) monitoring is considered the clinical reference standard for estimating P_mus_^7,8^ by quantifying changes in pleural pressure generated by the patient’s respiratory muscles. In patients with acute hypoxemic respiratory failure, higher inspiratory effort measured early by esophageal manometry has been associated with an increased risk of noninvasive ventilation failure^9^. Although physiologically robust, esophageal pressure monitoring is invasive, requires technical expertise, and has not been widely adopted in routine clinical practice. As a result, inspiratory effort is frequently assessed using surrogates such as airway occlusion pressure at 100 ms (P0.1), occlusion pressure assessed during airway occlusion for an entire breath (P_occ_), tidal volume, or visual waveform inspection^10,11^. However, these indices do not provide continuous quantification of inspiratory muscle pressure or allow direct calculation of work of breathing during assisted ventilation.

The equation of motion of the respiratory system, based on a simple one-compartment model of respiratory mechanics incorporating one value of airflow resistance (R_rs_) and one value of respiratory system elastance (E_rs_), provides a framework linking airway pressure (P_aw_), flow (V’), volume (V), and P_mus_^7,12,13^. Previous approaches have successfully exploited this relationship for reconstructing patient effort non-invasively by first estimating E_rs_ and R_rs_ in conditions of near-relaxation and subsequently employing the same values during assisted mechanical ventilation with relevant patient effort^14^. Their applicability during assisted ventilation is nevertheless limited by the need for assessment of R_rs_ and E_rs_ with minimal patient activity^13,14^. E_rs_ during assisted ventilation can be determined with sufficient accuracy by performing an end-inspiratory occlusion maneuver^15,16^, but reliable assessment of R_rs_ during assisted ventilation remains elusive.

In 2012, Ranieri et al. proposed a method for assessment of R_rs_ based on linear extrapolation of a P0.1 maneuver for determination of P_mus_ during early inspiration^17^. The present study was intended to evaluate a similar P0.1-guided, non-invasive method for estimating R_rs_ and, in conjunction with E_rs_ as obtained from an end-inspiratory occlusion maneuver, calculating P_mus_ and WOB during assisted mechanical ventilation. Conducted as a physiological substudy of the observational ICEBERG trial^18^, this study aimed to investigate the proposed algorithm in comparison to esophageal pressure–derived reference measurements in patients with acute hypoxemic respiratory failure. We hypothesized that P_mus_ and WOB reconstructed using this approach would show acceptable agreement with esophageal pressure-based measurements under clinically relevant conditions of assisted ventilation.

## Methods

### Study design and setting

This prospective physiological validation study was conducted as a substudy of the ICEBERG trial^18^, an observational study in critically ill patients undergoing assisted mechanical ventilation. The ICEBERG-trial was pre-registered at clinicaltrials.gov (NCT05203536). This hypothesis-generating substudy was not registered in a clinical trial registry.

The study was performed in the surgical intensive care units of University Medical Center Schleswig–Holstein, Campus Kiel. The protocol was approved by the local ethics committee (Ethikkommission der Medizinischen Fakultät der Christian-Albrechts-Universität zu Kiel, Kiel, Germany; Approval No. D573/21). As all ventilatory maneuvers performed as part of this study were part of routine clinical care and no study-specific procedures were applied, the ethics committee approved the study without the requirement for study-specific informed consent. All patients or their legal guardians provided written informed consent for the use and publication of their clinical data for research purposes. The study was carried out in accordance with all relevant guidelines and regulations.

### Study population

Adult patients (≥ 18 years) with AHRF, including patients fulfilling the Berlin definition of acute respiratory distress syndrome, were eligible if they were invasively mechanically ventilated in an assisted mode with preserved spontaneous breathing.

For inclusion in this substudy, high-quality recordings of airway pressure, flow, and volume as well as valid esophageal pressure measurements were required. Patients were excluded if esophageal balloon placement was contraindicated, if relevant air leaks or severe patient–ventilator asynchrony were present, or if signal quality did not allow analysis of airway plateau pressure for assessment of E_rs_^19^.

### Ventilatory management and signal acquisition

Patients were ventilated using the Elisa 800 VIT (Löwenstein Medical, Bad Ems, Germany) intensive care ventilators in assisted ventilation modes allowing spontaneous breathing, including pressure support ventilation and biphasic positive airway pressure. Ventilator settings such as pressure support level, positive end-expiratory pressure (PEEP), trigger sensitivity, and cycling criteria were determined by the treating clinicians and were not modified for study purposes.

P_aw_, P_es_, V’, and V were continuously recorded by the ventilator with a sampling rate of 200 Hz and stored for offline analysis. P_es_ was measured using a commercially available balloon catheter positioned in the lower third of the esophagus. Balloon filling and positioning were performed according to established recommendations and verified using standard occlusion tests^20^.

### P0.1 maneuver and data selection

Standardized end-expiratory airway occlusion maneuvers (P0.1 maneuvers) were performed to assess inspiratory drive and to enable non-invasive estimation of inspiratory muscle pressure. At end-expiration, the inspiratory valve was occluded for 100 ms, preventing airflow at the onset of inspiration. Assisted breaths immediately following valid P0.1 maneuvers were selected for further analysis.

Additionally, an end-inspiratory occlusion of 2 s duration was performed for assessment of E_rs_ as described by Foti et al.^15^. An end-inspiratory occlusion was considered readable when, after the onset of zero flow, a short and steep pressure rise was followed by the rapid attainment (typically < 800 ms) of a clearly identifiable flat plateau that remained stable for > 2 s, with minimal airway pressure drift (< 0.6 mbar/s)^19^. Tracings showing a slow, progressive pressure increase, a curved airway pressure profile, or the absence of a sustained flat segment were classified as unreadable, suggesting ongoing respiratory muscle activity.

### Step 1: Extrapolation of inspiratory muscle pressure from the P0.1 maneuver

**Fig. 1:**
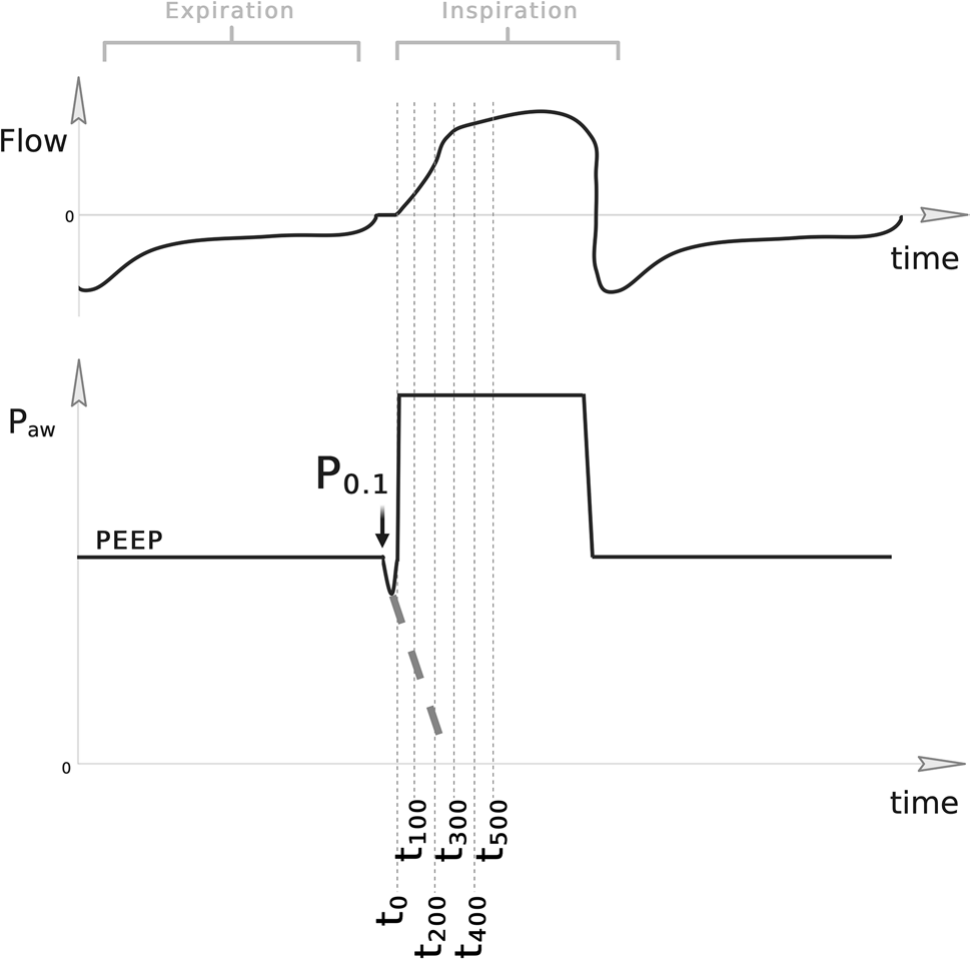
Method for determination of extrapolated inspiratory muscle pressure (P_mus,extrapolated_). Following a standardized 100-ms end-expiratory occlusion (P0.1 maneuver), the decrease in airway pressure under zero-flow conditions is linearly extrapolated into early inspiration. P_mus,extrapolated_ is calculated as the difference between the extrapolated airway pressure trajectory and the applied PEEP at predefined time points between 50 and 500 ms after the end of occlusion (50 ms increments). Selected time points (t0, t100, t200, t300, t400, and t500) are illustrated. Respiratory system resistance (R_rs_) can subsequently be estimated during early inspiration by inserting P_mus,extrapolated_ into the equation of motion and relating the resistive pressure component to the corresponding inspiratory flow. Created in BioRender. Behmüller, F. (2026) https://BioRender.com/3g86td1.

The first step of the method consisted of estimating inspiratory muscle pressure during the early phase of inspiration by extrapolating the airway pressure decrease observed during the P0.1 maneuver. For each maneuver, a linear regression was fitted to the airway pressure signal during the 100 ms occlusion interval. This regression line was extrapolated beyond the end of the occlusion into the early inspiratory phase (Fig. 1).

The extrapolation was performed up to predefined time points ranging from 50 ms to a maximum of 500 ms after the end of the P0.1 maneuver, in increments of 50 ms. At each time point, inspiratory muscle pressure was calculated as the difference between the extrapolated airway pressure trajectory (representing the hypothetical pressure under continued occlusion) and the positive end-expiratory airway pressure value prior to the P0.1 maneuver. This yielded a time-dependent, non-invasive estimate of inspiratory muscle pressure (P_mus,extrapolated_) during early inspiration.

### Step 2: Determination of airway resistance during early inspiration

Airway resistance was determined during early inspiration using the extrapolated inspiratory muscle pressure derived from the P0.1 maneuver. Resistance calculation was based on the equation of motion of the respiratory system:

$${\mathrm{P}}_{{{\mathrm{aw}}}} \left( {\mathrm{t}} \right)\, = \,{\mathrm{P}}_{{{\mathrm{mus}}}} \left( {\mathrm{t}} \right)\, + \,{\mathrm{R}} \cdot {{{\rm V}^{\prime}}} \left( {\mathrm{t}} \right)\, + \,{\mathrm{E}} \cdot {\mathrm{V}}\left( {\mathrm{t}} \right)\, + \,{\mathrm{PEEP}}.$$

where P_aw_(t) is the airway pressure at time t; P_mus_(t) is the pressure generated by the respiratory muscles; R is the respiratory system resistance; V’(t) is the inspiratory flow; E is the respiratory system elastance; V(t) is the volume of gas inspired above the end-expiratory level; PEEP is the positive end-expiratory pressure; and t is the time elapsed from the onset of inspiration.

All calculations were performed within the early inspiratory phase (≤ 500 ms after termination of the P0.1 occlusion), where the extrapolated inspiratory muscle pressure (P_mus,extrapolated_) was assumed to reflect the instantaneous patient-generated pressure.

Two different approaches were applied:

1. Elastance-uncorrected resistance

In the uncorrected approach, the resistive pressure component was calculated as:

$${\mathrm{P}}_{{{\mathrm{res}},{\mathrm{uncorr}}}} \left( {\mathrm{t}} \right) \, = \, \left( {{\mathrm{P}}_{{{\mathrm{aw}}}} \left( {\mathrm{t}} \right) \, {-}{\text{ PEEP}}} \right) \, + \, \left| {{\mathrm{P}}_{{{\mathrm{mus}},{\mathrm{extrapolated}}}} \left( {\mathrm{t}} \right)} \right|$$

No subtraction of the elastic pressure term was performed. Airway resistance was then obtained as: |

$${\mathrm{R}}_{{{\mathrm{rs}},{\mathrm{uncorr}}}} \, = {\text{ P}}_{{{\mathrm{res}},{\mathrm{uncorr}}}} \left( {\mathrm{t}} \right) \, / {{{\rm V}^{\prime}}} \left( {\mathrm{t}} \right)$$

This method therefore implicitly assumes that the elastic pressure contribution is negligible during early inspiration.

2. Elastance-corrected resistance

Even within the first 500 ms of inspiration, a small but arguably non-negligible volume has already been inhaled, generating an elastic recoil pressure (E·V(t)). If this elastic component is not subtracted, it artificially inflates the estimated resistive driving pressure and, consequently, the calculated resistance. The corrected approach therefore explicitly removes this elastic pressure term before calculating R_rs_, isolating the purely resistive component of the airway pressure signal. For this purpose, respiratory system elastance (E) was derived from inspiratory occlusion maneuvers. The elastic pressure component during inspiration was calculated as:

$${\mathrm{P}}_{{{\mathrm{el}}}} \left( {\mathrm{t}} \right) \, = {\text{ E}} \cdot {\mathrm{V}}\left( {\mathrm{t}} \right)$$

The corrected resistive pressure was then obtained as:

$${\mathrm{P}}_{{{\mathrm{res}},{\mathrm{corr}}}} \left( {\mathrm{t}} \right) \, = \, \left( {{\mathrm{P}}_{{{\mathrm{aw}}}} \left( {\mathrm{t}} \right) \, {-}{\text{ PEEP}}} \right) \, + \, \left| {{\mathrm{P}}_{{{\mathrm{mus}},{\mathrm{extrapolated}}}} \left( {\mathrm{t}} \right)} \right| \, - {\text{ P}}_{{{\mathrm{el}}}} \left( {\mathrm{t}} \right)$$

Airway resistance was subsequently calculated as:

$${\mathrm{R}}_{{{\mathrm{rs}},{\mathrm{corr}}}} = {\text{ P}}_{{{\mathrm{res}},{\mathrm{corr}}}} \left( {\mathrm{t}} \right)/ {{{\rm V}^{\prime}}} \left( {\mathrm{t}} \right)$$

Both resistance estimates were compared with reference resistance values derived from esophageal pressure–based measurements by least squares fitting^21^. In addition, the inspiratory occlusion-based elastance values (E_occ_), that were used for calculation of R_rs,corr_ as well as subsequent parameterization of the equation of motion (see below), were compared to esophageal-pressure based reference values (E_ref_) obtained by least squares fitting. This reference approach assumes a single-compartment, linearly behaving respiratory system with constant resistance and elastance over the analysed breath and negligible inertance, and that esophageal pressure faithfully reflects pleural pressure; correct esophageal catheter position was confirmed by an occlusion (Baydur) test.

### Step 3: Parameterization of the equation of motion and reconstruction of inspiratory muscle pressure and work of breathing

After determination of R and E, the respiratory system equation of motion was parameterized as:

$${\mathrm{P}}_{{{\mathrm{aw}}}} \left( {\mathrm{t}} \right) \, = {\text{ P}}_{{{\mathrm{mus}}}} \left( {\mathrm{t}} \right) \, + {\text{ R}} \cdot {{{\rm V}^{\prime}}} \left( {\mathrm{t}} \right) \, + {\text{ E}} \cdot {\mathrm{V}}\left( {\mathrm{t}} \right) \, + {\text{ PEEP}}$$

For further reconstruction of inspiratory muscle pressure over the entire breath, the equation was rearranged:

$${\mathrm{P}}_{{{\mathrm{mus}},{\mathrm{calc}}}} \left( {\mathrm{t}} \right) \, = {\text{ P}}_{{{\mathrm{aw}}}} \left( {\mathrm{t}} \right) \, - {\text{ R}} \cdot {{{\rm V}^{\prime}}} \left( {\mathrm{t}} \right) \, - {\text{ E}} \cdot {\mathrm{V}}\left( {\mathrm{t}} \right) \, - {\text{ PEEP}}$$

where P_mus,calc_(t) is the reconstructed inspiratory muscle pressure; all other terms are defined as above.

Using these individualized parameter estimates, the time-resolved inspiratory muscle pressure was reconstructed over the complete inspiratory phase. From the reconstructed pressure curve, maximal inspiratory muscle pressure, work of breathing (WOB), and pressure–time product (PTP) were subsequently calculated.

WOB was calculated as the integral of inspiratory muscle pressure over tidal volume and expressed in joules per breath. In addition, WOB normalized to tidal volume (J/L) was calculated. PTP was calculated as the time integral of inspiratory muscle pressure over the inspiratory phase and expressed in mbar·s/min.

All parameters were independently calculated from esophageal pressure measurements using established methods and served as reference values for validation.

### Statistical analysis

Agreement between non-invasive estimates and esophageal pressure–derived reference measurements was assessed using linear regression and Bland–Altman analysis. The coefficient of determination (R^2^), obtained from linear regression analysis, was used to describe the strength of association. For Bland–Altman analyses, bias was defined as the difference between the estimated parameter and the corresponding reference value, and 95% limits of agreement were calculated as bias ± 1.96 standard deviations of the differences.

Because multiple measurements were obtained from some patients, the primary analysis was performed using patient-level averaged values: all measurements obtained from the same patient were averaged so that each patient contributed a single data point (n = 18), thereby avoiding pseudo-replication and the imbalance arising from unequal numbers of measurements per patient. To assess robustness, a sensitivity analysis was additionally performed using all individual measurements analysed independently (n = 33).

Statistical significance was defined as a two-sided p value < 0.05. Continuous variables are reported as mean ± standard deviation unless stated otherwise.

## Results

### Study population

**Fig. 2:**
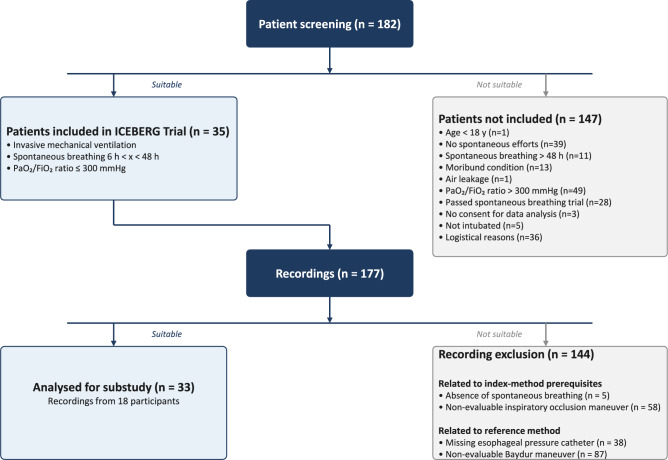
Patient selection and flow of recordings. Flow diagram of patient inclusion in the parent ICEBERG cohort and subsequent selection of evaluable recordings for this substudy. Recordings were excluded for reasons related either to prerequisites of the index method or to the esophageal-pressure reference standard. Note that the individual counts under "Patient not included" and “Recording exclusion” sum to more than the respective totals, because several criteria applied simultaneously in some patients.

From the parent ICEBERG cohort at our center (n = 35), 33 datasets from 18 patients with acute hypoxemic respiratory failure were suitable for inclusion in this substudy after quality control (Fig. 2). Participants had a mean age of 67 ± 12 years, a body weight of 88 ± 19 kg, and height of 176 ± 10 cm. Five patients (28%) were female and thirteen (72%) male. Seven patients (39%) fulfilled criteria for acute respiratory distress syndrome, while eleven (61%) were classified as acute hypoxemic respiratory failure without ARDS. The mean PaO₂/FiO₂ ratio at study inclusion was 198 ± 44 mmHg. Sedation depth assessed using the Richmond Agitation and Sedation Scale was − 1.5 (Median; Interquartile Range: − 4 to − 0.75). Baseline patient characteristics are summarized in Table 1.

**Table 1:** Demographic and clinical characteristics of patients included in the analysis.

Variable	Value (mean ± SD)
Age (years)	67 ± 12
Male / Female (%)	72 / 28
PaO₂/FiO₂ (mmHg)	198 ± 44
FiO₂ (%)	42 ± 10
PEEP (mbar)	9.6 ± 3.3
ΔP_insp_ (mbar)	8.1 ± 2.7
V_T,e_ (ml)	589 ± 140
Respiratory rate (1/min)	18 ± 6
Ventilator mode (number / % of recordings)	27 (82%) PSV; 5(15%) BILEVEL; 1(3%) WOBOV
Etiology of AHRF (number / % of patients in substudy)	Pneumonia: 3 (17%); CPR: 3 (17%); myocardial infarction: 2 (11%); non-pulmonary sepsis: 5 (28%); trauma or major surgery: 3 (17%); other: 2 (11%)

Abbreviations: AHRF = Acute Hypoxemic Respiratory Failure; CPR = Cardiopulmonary Resuscitation; FiO_2_ = inspired fraction of oxygen; PaO_2_ = arterial partial pressure of oxygen; PEEP = positive end-expiratory pressure; ΔP_insp_ = inspiratory pressure support (for PSV) or set inspiratory pressure difference (for BILEVEL); V_T,e_ = expired tidal volume; PSV = pressure support ventilation; BILEVEL = Biphasic Positive Airway Pressure; WOBOV = Work of Breathing Optimized Ventilation.

### Non-invasive estimation of inspiratory muscle pressure

Non-invasive estimation of P_mus,extrapolated_ by linear extrapolation of the airway pressure decrease during the P0.1 maneuver into early inspiration succeeded in all patients. Inspiratory muscle pressure was calculated at predefined time points between 50 and 500 ms after the P0.1 occlusion.

**Fig. 3:**
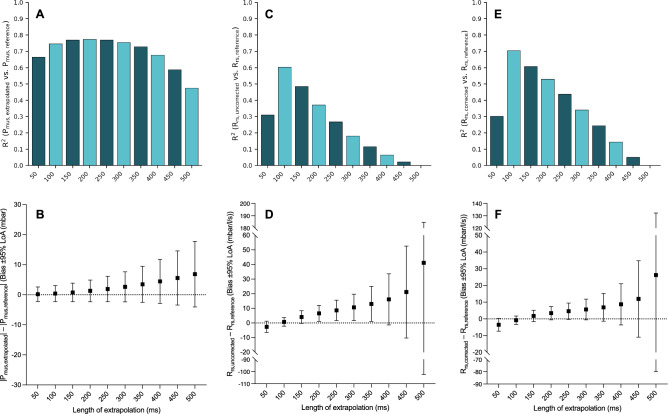
Agreement between non-invasive estimates and esophageal pressure–derived reference values as a function of extrapolation length after the P0.1 maneuver. Upper panels display coefficients of determination (R^2^), and lower panels corresponding bias and 95% limits of agreement (LoA) derived from Bland–Altman analysis. Panels **A, B**: inspiratory muscle pressure estimated by extrapolation (P_mus,extrapolated_) versus reference P_mus_. Panels **C, D**: uncorrected airway resistance (R_rs,uncorr_) versus reference resistance (R_rs,ref_). Panels **E, F**: elastance-corrected airway resistance (R_rs,corr_) versus R_rs,ref_. Agreement for R_rs_ was highest at 100 ms extrapolation time and progressively declined with longer extrapolation intervals, accompanied by increasing systematic bias. Note: y-axes in Panels D and F are broken to accommodate extreme bias values at extrapolation times ≥ 450 ms. All analyses are based on patient-averaged values (primary analysis; one value per patient, n = 18).

Across all time points between 50 and 500 ms, P_mus,extrapolated_ correlated with esophageal pressure–derived reference values (all p < 0.01). The strength of agreement depended on the timing of extrapolation. The highest coefficients of determination (R^2^) were observed between 100 and 350 ms after the P0.1 maneuver. The lowest bias was observed during early inspiration, with increasing bias with longer durations of extrapolation. Coefficients of determination and biases for all extrapolation times are summarized in Fig. 3 (panels A and B).

At 100 ms after P0.1, which was selected as a clinically relevant compromise between early extrapolation and signal stability, the estimated inspiratory muscle pressure demonstrated good agreement with the reference method (R^2^ = 0.75) and a low bias of 0.39 mbar (95% limits of agreement − 2.3 to + 3.1 mbar).

### Estimation of airway resistance

Airway resistance could be calculated in all analyzed breaths using both elastance-corrected as well as uncorrected approaches.

#### Elastance-uncorrected resistance

For uncorrected resistance, statistically significant correlations with reference values were observed for lengths of extrapolation between 50 and 250 ms (all p < 0.05). The highest agreement was found at 100 ms extrapolation after P0.1, with R^2^ = 0.60 and minimal bias of + 0.74 mbar/l/s (95% limits of agreement − 2.2 to + 3.7 mbar/l/s). Beyond 250 ms, correlations were no longer statistically significant. Results for uncorrected R_rs_ are summarized in Fig. 3, Panels C and D.

#### Elastance-corrected resistance

Elastance-corrected resistance showed improved agreement compared with uncorrected resistance. At 100 ms after P0.1, corrected resistance achieved an R^2^ of 0.70 with a low bias of − 0.78 mbar/l/s.

Across all evaluated time points, elastance correction consistently reduced systematic error and narrowed limits of agreement. Results for elastance-corrected R_rs_ are presented in Fig. 3, Panels E and F. The complete individual regression and Bland–Altman plots for all three parameters at each extrapolation duration are provided as Supplementary Figs. S1 (patient-averaged primary analysis, n = 18) and S2 (per-measurement sensitivity analysis, n = 33).

#### Elastance determined using inspiratory hold maneuvers

Values of E_occ_ determined using the inspiratory hold maneuver showed moderate agreement with reference values obtained from esophageal pressure measurements by least squares fitting, with R^2^ of 0.42 and bias of − 3.5 mbar/l.

**Fig. 4:**
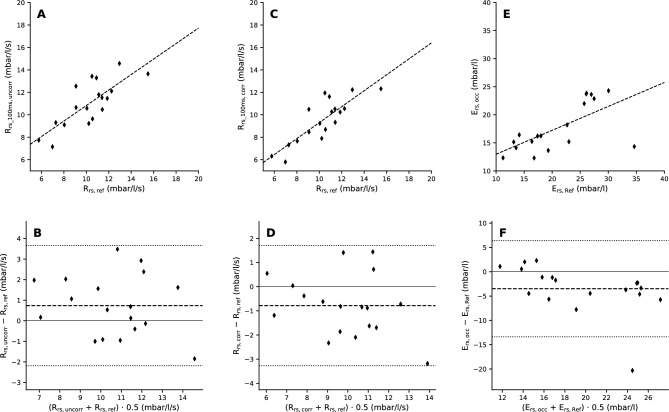
Agreement between non-invasive estimates of airway resistance and respiratory system elastance with esophageal pressure–derived reference values at an extrapolation time of 100 ms. Upper panels display coefficients of determination (R^2^), and lower panels corresponding bias and 95% limits of agreement (LoA) derived from Bland–Altman analysis. Panels **A, B**: uncorrected airway resistance (R_rs,uncorr_) in comparison reference values (R_rs,ref_) derived from esophageal pressure measurements by least squares fitting; Panels **C, D**: elastance-corrected airway resistance (R_rs,corr_) in comparison to R_rs,ref_; Panels **E, F**: inspiratory occlusion-derived elastance (E_occ_) versus in comparison reference values (E_ref_) derived from esophageal pressure measurements by least squares fitting. In the Bland–Altman plots, the solid line represents the mean bias and the dotted lines indicate the 95% limits of agreement. All analyses are based on patient-averaged values (primary analysis, n = 18).

Results for both uncorrected and elastance-corrected values of R_rs_ using an extrapolation time of 100 ms as well as E_occ_ in comparison to reference values are detailed in Fig. 4.

### Inspiratory muscle pressure derived from the equation of motion

Inspiratory muscle pressure reconstructed using the equation of motion (P_mus,calc_), parameterized with elastance-corrected R_rs_ derived at an extrapolation time of 100 ms and E_occ_, demonstrated good agreement with esophageal pressure–derived reference values (P_mus,es_). Linear regression analysis yielded an R^2^ of 0.66 (p < 0.0001). Bland–Altman analysis revealed a small bias of − 0.99 mbar with 95% limits of agreement from − 5.91 to + 3.93 mbar.

**Fig. 5:**
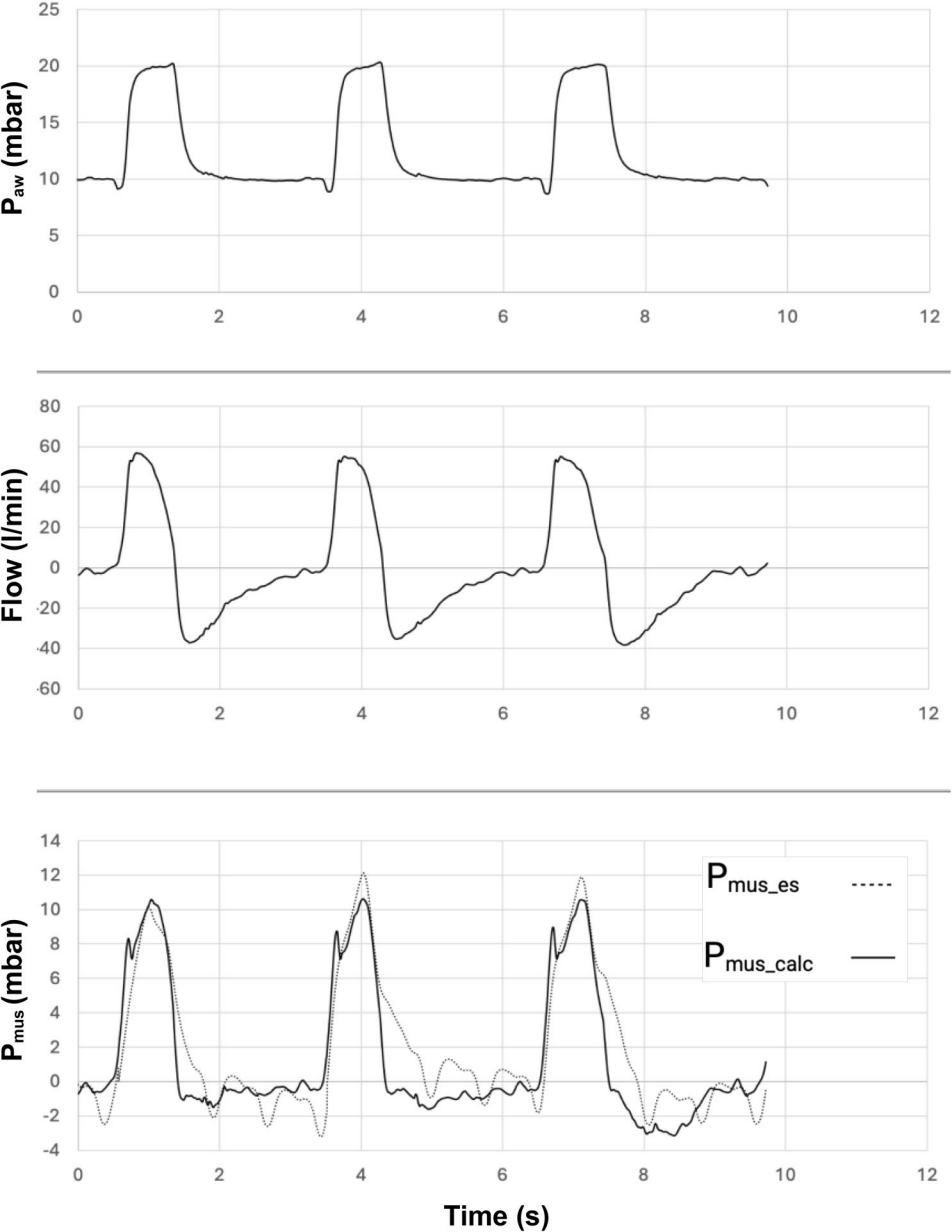
Representative time-course of airway pressure, inspiratory flow, and inspiratory muscle pressure during assisted mechanical ventilation. The upper panel shows airway pressure (P_aw_), the middle panel inspiratory flow, and the lower panel inspiratory muscle pressure derived from esophageal measurements (P_mus,es_, dotted line) and reconstructed non-invasively using the equation of motion (P_mus,calc_, solid line). The reconstructed pressure waveform closely follows the reference signal throughout the inspiratory phase, illustrating the feasibility of dynamic P_mus_ estimation by incorporating P0.1-derived resistance and inspiratory occlusion-derived elastance into the equation of motion. Differences during the expiratory phase are expected, as the method is not designed to reconstruct expiratory muscle activity.

A representative example of the temporal course of P_mus,calc_ in comparison with P_mus,es_ is shown in Fig. 5.

### Work of breathing

Work of breathing calculated from P_mus,calc_ showed good agreement with esophageal pressure–derived reference values. For work of breathing expressed in joules per breath, linear regression demonstrated a strong correlation (R^2^ = 0.70, p < 0.0001). Bland–Altman analysis showed a minimal bias of − 0.05 J with narrow 95% limits of agreement (− 0.27 to + 0.16 J).

When normalized to tidal volume (J/L), work of breathing remained strongly correlated with reference values (R^2^ = 0.71, p < 0.0001), with a bias of − 0.09 J/L and wider limits of agreement (− 0.43 to + 0.25 J/L).

### Pressure–time product

Pressure–time product calculated based on P_mus,calc_ showed a good correlation with esophageal pressure–derived values (R^2^ = 0.62, p < 0.001). Bland–Altman analysis revealed a bias of − 8.58 mbar·s/min and 95% limits of agreement ranging from − 58.3 to + 41.1 mbar·s/min.

**Fig. 6:**
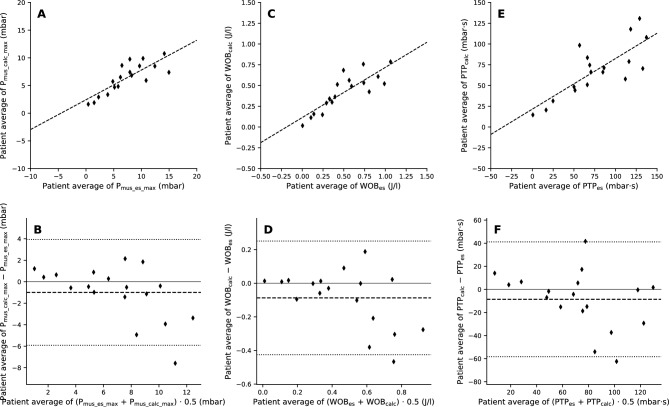
Agreement between non-invasively reconstructed effort-related parameters and esophageal pressure–derived reference values. Upper panels show linear regression analyses for maximal inspiratory muscle pressure (P_mus,max_; **A**), work of breathing (WOB; **C**), and pressure–time product (PTP; **E**). Lower panels display the corresponding Bland–Altman analyses for P_mus,max_ (**B**), WOB (**D**), and PTP (**F**). In the Bland–Altman plots, the solid line represents the mean bias and the dotted lines indicate the 95% limits of agreement. P_mus,max_ and WOB demonstrated acceptable agreement with the reference method, whereas PTP showed wider limits of agreement and greater variability. All analyses are based on patient-averaged values (primary analysis, n = 18).

Linear regression and Bland–Altman plots for P_mus,calc_ as well as WOB and PTP are shown in Fig. 6.

### Sensitivity analyses

**Fig. 7:**
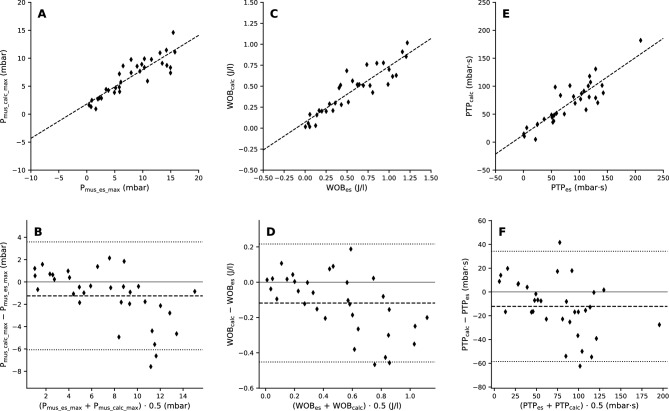
Agreement between non-invasively reconstructed effort-related parameters and esophageal pressure–derived reference values based on all individual measurements analysed independently (sensitivity analysis). Upper panels show linear regression analyses for maximal inspiratory muscle pressure (P_mus,max_; **A**), work of breathing (WOB; **C**), and pressure–time product (PTP; E), comparing all individual measurements derived from the proposed method with corresponding esophageal pressure–derived reference values. Lower panels display the corresponding Bland–Altman analyses for P_mus,max_ (**B**), WOB (**D**), and PTP (**F**). In the Bland–Altman plots, the solid line represents the mean bias and the dotted lines indicate the 95% limits of agreement. Data points represent all individual measurements analysed independently (n = 33).

When analyses were repeated using all individual measurements analysed independently (n = 33), the overall agreement between reconstructed parameters and esophageal pressure–derived reference values remained comparable to the primary, patient-averaged analysis, although the limits of agreement tended to widen, with larger differences observed at higher values for some parameters (Fig. 7).

## Discussion

In this physiological substudy of the ICEBERG observational trial, we investigated a novel non-invasive approach for estimating inspiratory P_mus_, R_rs_, WOB and PTP during assisted mechanical ventilation in patients with acute hypoxemic respiratory failure. The principal finding is that P_mus_ derived from linear extrapolation of the airway pressure signal following a P0.1 maneuver shows acceptable agreement with esophageal pressure–based reference measurements, particularly within the time window of 100 – 150 ms after the end of the P0.1 occlusion. During this time period, it can serve as a basis for calculation of R_rs_ using the corresponding flow signal during the early phase of inspiration. By combining R_rs_ obtained from this method and E_rs_ obtained during an end-inspiratory occlusion^15,19^, clinically relevant secondary parameters like maximal inspiratory P_mus_ and WOB could be estimated with acceptable accuracy. Estimation of PTP was less accurate.

### Validity of P0.1-guided extrapolation of inspiratory muscle pressure

The estimation of inspiratory muscle pressure based on extrapolation of the airway pressure drop during a P0.1 maneuver proved feasible in all included patients. Agreement with esophageal pressure–derived reference values depended on the timing of extrapolation, with the best compromise between low bias and high coefficient of determination obtained 100–150 ms after the occlusion and a progressive decline at later time points. This temporal pattern is physiologically plausible. Early after the occlusion, the pressure signal predominantly reflects the initial inspiratory muscle activation against a closed system, whereas later during inspiration, changes in respiratory system mechanics, flow-dependent resistive components, and dynamic recruitment of respiratory muscles increasingly influence the pressure trajectory. These factors likely violate the assumption of linearity underlying the extrapolation and explain the deterioration of agreement at later time points.

From a clinical perspective, the results obtained at 100 ms after P0.1 are particularly relevant. At this early time point, agreement with the reference method was acceptable, bias was low, and signal quality was high. This timing aligns with the physiological concept of P0.1 as a measure of early inspiratory drive and suggests that short occlusion-based extrapolation captures a meaningful and reproducible component of inspiratory muscle pressure. Importantly, this approach requires no additional hardware beyond standard ventilator measurements and a brief, well-established occlusion maneuver.

### Estimation of airway resistance

Using the non-invasively estimated inspiratory muscle pressure as a starting point, airway resistance could be calculated throughout assisted inspiration. Uncorrected resistance, neglecting the influence of elastic recoil during early inspiration, showed acceptable agreement with reference values, whereas correction for elastic recoil of the respiratory system led to slightly improved agreement, with a higher coefficient of determination and narrower limits of agreement. Again, using P_mus,extrapolated_ at 100 ms after P0.1 in conjunction with the corresponding flow resulted in the best agreement and lowest bias for R_rs_ in comparison to the P_es_-derived reference values obtained by least squares fitting. Despite its inferior agreement with reference values, the elastance-uncorrected approach retains practical relevance and was therefore retained as a comparator in this analysis. Elastance correction requires a readable end-inspiratory plateau pressure, which presupposes sufficient relaxation of the respiratory muscles during the end-inspiratory occlusion maneuver. While all measurements included in the present study met this criterion, recordings with non-readable plateaus accounted for a substantial proportion of excluded measurements in our cohort. In these patients, who may carry the highest burden of inspiratory effort and thus arguably represent the population in greatest need of non-invasive monitoring, determination of E_occ_ and consequently R_rs,corr_ is not feasible. The uncorrected resistance estimate, requiring only the P0.1 maneuver and the corresponding flow signal, remains applicable in this subgroup and may still provide physiologically meaningful information on airway mechanics and inspiratory effort, albeit with reduced precision.

### Inspiratory muscle pressure derived from the equation of motion

P_mus_ reconstructed for the whole duration of inspiration using the equation of motion demonstrated acceptable agreement with esophageal pressure measurements, with a small negative bias (slight underestimation of the reference). This result indicates that, once respiratory system mechanics are reasonably characterized, dynamic reconstruction of the inspiratory muscle pressure waveform is feasible using E_rs_ and R_rs_ determined with the described method. However, the observed bias and variability also suggest that simplifications inherent to the equation of motion, such as assumptions of linear elastance and resistance and neglect of inertance with our approach, limit precision.

### Work of breathing and pressure–time product

Among all derived parameters, work of breathing showed the strongest agreement with esophageal pressure–based reference measurements. Bias was minimal and limits of agreement were narrow, both for absolute work of breathing and for work normalized to tidal volume. This robustness likely reflects the integrative nature of work of breathing, which averages local discrepancies in instantaneous pressure estimation over the entire inspiratory volume. As a result, small temporal deviations in the reconstructed pressure curve have limited impact on the final value.

In contrast, pressure–time product showed greater bias and wider limits of agreement. This observation is consistent with the fact that pressure–time product is highly sensitive to the precise temporal evolution of inspiratory muscle pressure, including isometric phases at the onset of inspiration. Any temporal smoothing or misalignment introduced by extrapolation or waveform reconstruction disproportionately affects this metric. Consequently, while pressure–time product remains physiologically meaningful, work of breathing appears to be the more robust and clinically useful parameter when derived from non-invasive pressure estimation.

### Clinical implications

These findings have several potential clinical implications. First, the proposed method enables estimation of R_rs_, E_rs_ and effort-related parameters in patients undergoing assisted mechanical ventilation without esophageal pressure monitoring or the need for full-breath occlusion maneuvers^11^, providing a potentially less-invasive alternative for assessment of patient effort. Second, continuous or repeated non-invasive quantification of work of breathing could support titration of pressure support, helping to avoid both excessive inspiratory effort, which may contribute to patient self-inflicted lung and diaphragm injury, and insufficient effort, which may promote diaphragmatic disuse.

Recently, complete airway occlusion has been proposed as an alternative method for estimation of P_mus_ and transpulmonary pressure during assisted mechanical ventilation^22^. By measuring the total airway pressure swing generated against a fully occluded airway (ΔP_occ_), scalar estimates of P_mus_ and dynamic transpulmonary driving pressure can be obtained using fixed conversion factors. While this approach is simple and requires no additional equipment, it provides a single per-breath scalar value rather than a time-resolved pressure waveform, and therefore does not directly enable calculation of work of breathing. The P0.1-guided method evaluated in the present study is conceptually complementary: rather than capturing a single pressure swing under full occlusion, it uses brief end-expiratory occlusion in combination with equation-of-motion modeling to reconstruct the continuous inspiratory P_mus_ profile, from which integrative parameters such as WOB can be derived.

### Limitations

Several limitations must be acknowledged. The study was conducted as a substudy within a larger observational trial, resulting in a relatively small and preselected patient cohort. Notably, only 33 of 177 recordings (19%) were ultimately analysable; that is, more recordings were excluded than analysed, which bears on the applicability of the technique. The exclusions fell into two categories with distinct implications (Fig. 2). The larger category was related to the esophageal pressure–based reference standard and is specific to the present validation setting: no esophageal balloon catheter was in place (n = 38), or the catheter position could not be validated because the Baydur (end-expiratory occlusion) test was non-evaluable, so that the esophageal pressure signal was unusable as a reference (n = 87). These reference-related exclusions would not arise in clinical use, where the proposed method is intended precisely to render esophageal pressure measurement unnecessary. The second category is related to prerequisites of the index method itself: absence of spontaneous effort, leaving no inspiratory activity to analyse (n = 5), and a non-readable end-inspiratory occlusion plateau^19^ (n = 58). The latter warrants emphasis, because an unreadable plateau may occur preferentially in patients with vigorous inspiratory effort and incomplete muscle relaxation during the hold. In the ICEBERG multicenter cohort, the proportion of non-readable inspiratory plateaus was higher in non-survivors than in survivors. This implies that these patients potentially represent a particularly vulnerable subgroup in whom elastance correction and the non-invasive estimation of WOB and PTP via the parameterized equation of motion may be less reliably obtainable. As a consequence, our analyzable sample could be enriched for patients with less severely impaired respiratory mechanics, potentially limiting the external validity of our findings. The method is therefore expected to perform most reliably in patients with moderate inspiratory effort and adequate relaxation during the end-inspiratory hold, whereas its performance may be reduced in those with vigorous, sustained effort precluding a readable plateau. As noted above, however, the simplified uncorrected-resistance variant, which requires only the P0.1 maneuver and the corresponding flow, remains applicable in this subgroup and can still provide physiologically meaningful information, albeit with reduced precision. By contrast, the categories listed as ‘not included’ in Fig. 2 reflect the selection criteria of the parent ICEBERG cohort rather than limitations of the technique. Taken together, although the proportion of evaluable recordings in this validation study was limited, the real-world applicability of the method is likely to be broader; its feasibility and robustness in unselected, spontaneously breathing patients nevertheless remain to be confirmed prospectively.

The method relies on the performance of P0.1 maneuvers, which, although widely available, may not be feasible or reliable in all patients or ventilatory conditions. Furthermore, respiratory system mechanics were assumed to be linear over the analyzed range, and time-varying changes in compliance, resistance, or the influence of inertance as well as flow-dependent changes in resistance due to turbulence were not explicitly modeled. With respect to flow-dependent resistance, the resistive pressure of the respiratory system increases with flow (Rohrer behaviour). In patients with low-to-moderate inspiratory peak flows, the error introduced by assuming a constant linear inspiratory resistance is expected to be modest, but it may become more relevant in patients with high inspiratory drive and correspondingly high peak flows, as can occur in acute hypoxemic respiratory failure. Dynamic hyperinflation with intrinsic PEEP, although generally less pronounced in acute hypoxemic respiratory failure than in obstructive disease, may occur at high respiratory rates and short expiratory times; any unaccounted intrinsic PEEP would act as an elastic threshold load that is not captured by the single-compartment model referenced to the set PEEP and could bias the reconstructed inspiratory muscle pressure and work of breathing. We did not quantify these effects at the individual-patient level, and their explicit incorporation (e.g., flow-dependent resistance models or measurement of intrinsic PEEP) represents an avenue for future refinement.

In addition, the reference is itself model-based: esophageal pressure–derived values are the accepted physiological standard for inspiratory effort but not an absolute gold standard, and they share the single-compartment framework with the proposed method. Errors in the reference therefore add variability that would tend to attenuate rather than inflate the observed agreement. In contrast, the shared model structure, which is based on a one-compartment equation of motion framework, may contribute to some of the observed agreement. The results should be interpreted accordingly.

In keeping with the exploratory, hypothesis-generating design of this substudy, agreement was summarized using the standard parametric limits of agreement, the distributional assumptions of which were assessed by visual inspection of the difference plots rather than by formal normality testing. For the same reason, point estimates for bias, limits of agreement, and regression-based measures of agreement (R^2^) are reported without accompanying confidence intervals, which, given the modest sample size, would themselves carry considerable uncertainty and add little interpretative value. The resulting estimates should therefore be regarded as descriptive and indicative rather than as precise quantifications of agreement, warranting confirmation in larger, adequately powered cohorts. Finally, this study was purely physiological and not designed to assess clinical outcomes or to define thresholds for intervention.

## Conclusions

In conclusion, our preliminary results suggest that a P0.1-guided, non-invasive method based on extrapolation of airway pressure signals may allow feasible and physiologically plausible estimation of R_rs_ and P_mus_ during assisted mechanical ventilation. When combined with appropriate modeling of respiratory mechanics, this approach could allow non-invasive estimation of work of breathing and airway resistance without esophageal pressure monitoring. These findings support further evaluation of non-invasive inspiratory effort monitoring as a tool to individualize assisted ventilation and to better balance lung protection and diaphragmatic load in patients with acute hypoxemic respiratory failure.

## Supplementary Information

Supplementary Information 1.

Supplementary Information 2.

## Data Availability

Raw data will be made available from the corresponding author upon reasonable request.
