# Supplementary material for: Non-invasive estimation of inspiratory muscle pressure and work of breathing by airway pressure extrapolation from the P0.1 maneuver during assisted ventilation
Source: Sci Rep. 2026 Jul 16;16:22412. doi: 10.1038/s41598-026-61929-1 (PMC13377170; doi:10.1038/s41598-026-61929-1)

Supplementary Figure S2a — $P_{mus, extrap}$ across extrapolation durations — per-measurement sensitivity analysis (n = 33)

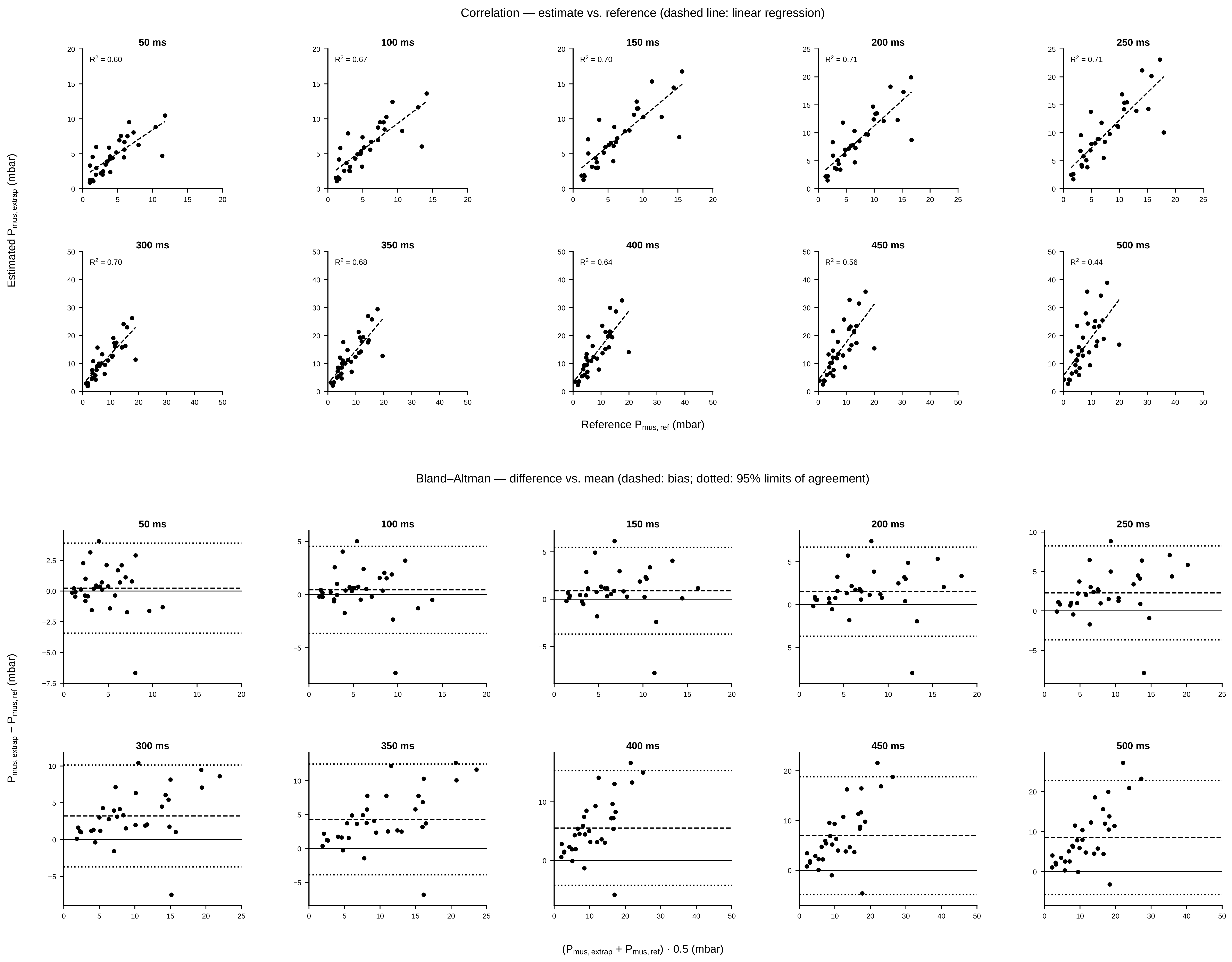

Supplementary Figure S2b — $R_{rs,\,uncorr}$ across extrapolation durations — per-measurement sensitivity analysis (n = 33)

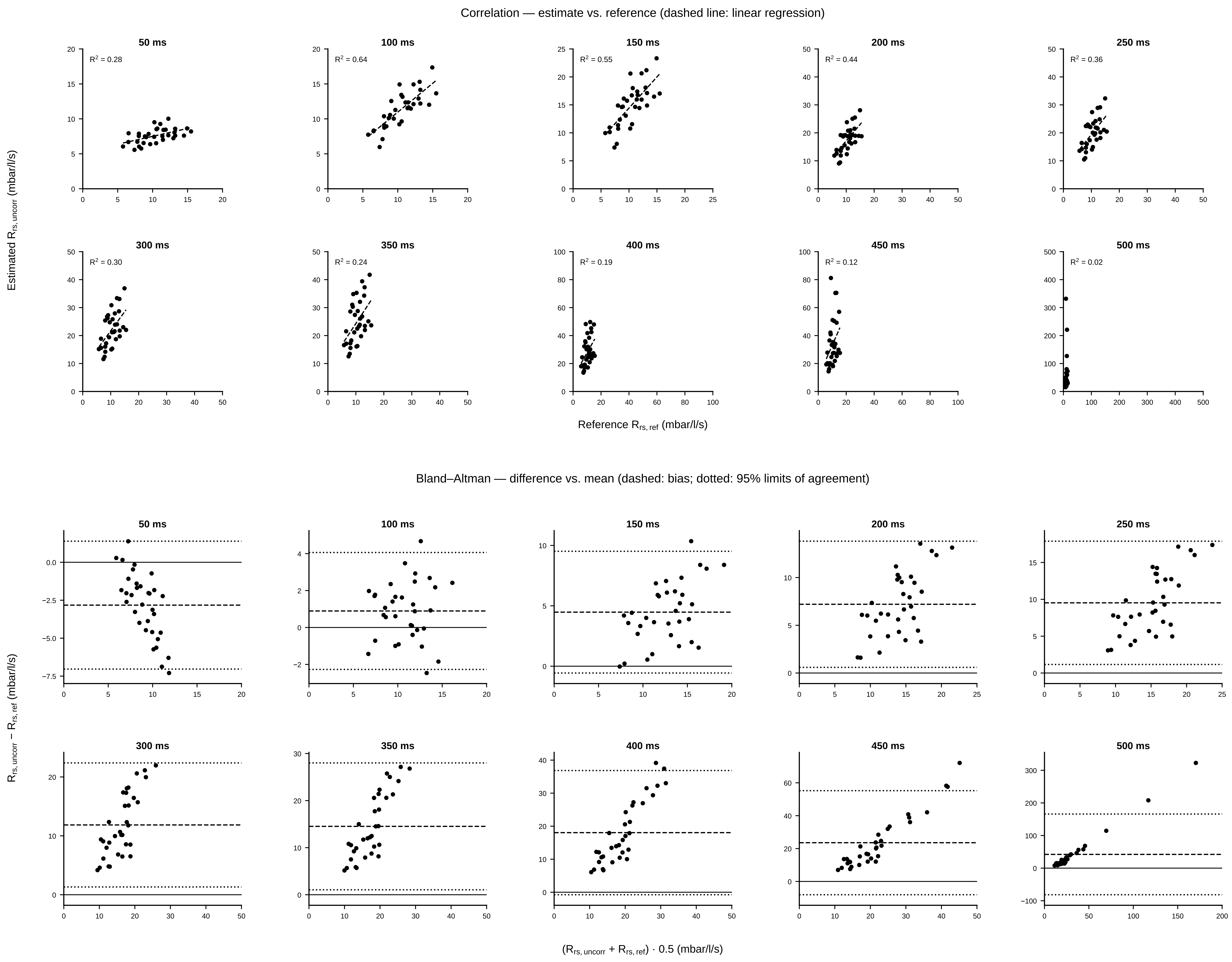

Supplementary Figure S2c — $R_{rs,corr}$ across extrapolation durations — per-measurement sensitivity analysis (n = 33)

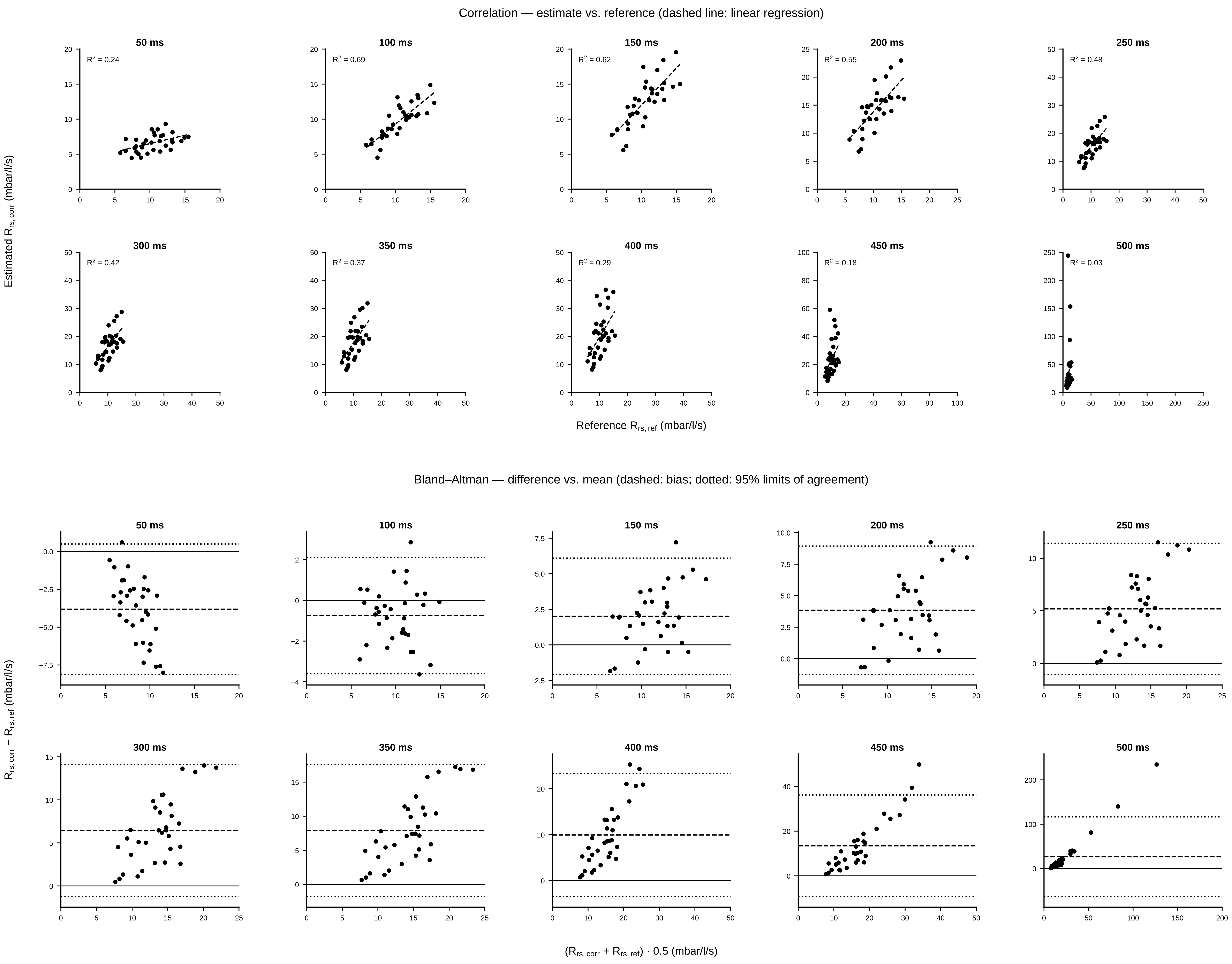

Supplement: Supplementary file 2 — Supplementary Information 2. [file 41598_2026_61929_MOESM2_ESM.pdf]
